# Acute isometric handgrip exercise enhances cardiac baroreflex sensitivity and lowers systolic blood pressure in chronic kidney disease

**DOI:** 10.1016/j.autneu.2026.103446

**Published:** 2026-06-10

**Authors:** Jeann L. Sabino-Carvalho, Alina Niu, Elsa Mekonnen, Jeanie Park

**Affiliations:** a Division of Renal Medicine, Department of Medicine, Emory University School of Medicine, Atlanta, GA, USA; b Department of Veterans Affairs Health Care System, Decatur, GA, USA; c Faculty of Physical Education, University of Brasília, Brasília, DF, Brazil

**Keywords:** Chronic kidney disease, Blood pressure, Baroreflex, Autonomic nervous system

## Abstract

Chronic kidney disease (CKD) impairs cardiac baroreflex sensitivity (cBRS), contributing to poor blood pressure (BP) control and heightened cardiovascular risk. Emerging evidence indicates that isometric handgrip (IHG) exercise may enhance cBRS in healthy individuals; however, its effects in CKD remain unclear. Therefore, this study tested the hypothesis that a single IHG session increases cBRS and reduces beat-to-beat BP variability (BPV) in CKD patients. In 21 patients (61 ± 12 yr; stages III-IV), beat-to-beat BP (finger photoplethysmography), heart rate (HR, electrocardiography), and respiration were continuously measured before and 10-, 20-, and 30-min post IHG vs. sham exercise in a crossover design. cBRS was assessed via the sequence technique and cardiac autonomic modulation via time- and frequency-domain HR variability (HRV). BPV was quantified using time domain indexes. cBRS increased following IHG exercise (10-min: Δ20 ± 4%; 20-min: Δ23 ± 5%; 30-min: Δ17 ± 5%; all *P* < 0.004 vs rest). This increase was significantly different from sham at 10-min and 20-min (all *P* < 0.006 vs sham), but not at 30-min post-IHG (*P* = 0.074). HR decreased throughout recovery (all *P* = 0.001 vs rest). Systolic BP decreased 30-min following IHG exercise (Δ-5 ± 2 mmHg; *P* = 0.047 vs. rest). Time-domain HRV increased during recovery (*P* = 0.005) whereas BPV remained unchanged following IHG exercise. After sham, all variables remained similar to rest, except systolic BP was significantly higher 20-min after sham. A single IHG session increased cBRS and vagal modulation, with a modest reduction in systolic BP and no change in BPV, in CKD patients. Overall, these findings suggest that IHG may serve as a non-pharmacological intervention for cardiovascular regulation in CKD.

## 1. Introduction

Chronic kidney disease (CKD) affects more than 850 million individuals worldwide and substantially increases the risk of cardiovascular morbidity and mortality (Matsushita et al., 2022). This elevated risk extends beyond sustained elevations in blood pressure (BP) to include abnormal fluctuations in BP, including exaggerated beat-to-beat BP variability (BPV) (Parati et al., 2018; Wei et al., 2014). Heightened BPV independently predicts left ventricular hypertrophy, arrhythmias, stroke, and premature death, emphasizing that instability of BP regulation is a clinically important but underexplored contributor to cardiovascular risk in CKD (Muntner et al., 2011; Tatasciore et al., 2007).

The arterial baroreflex plays a central role in buffering short-term fluctuations in BP (Sabino-Carvalho et al., 2021), and its impairment is consistently reported in CKD (Sabino-Carvalho et al., 2023b; Sabino-Carvalho et al., 2024; Tinucci et al., 2001). We recently demonstrated that patients with CKD not only present with reduced cardiac baroreflex sensitivity (cBRS) but also augmented beat-to-beat BPV (Sabino-Carvalho et al., 2023b), linking baroreflex dysfunction with abnormal BP dynamics (Cowley Jr. et al., 1973). These findings highlight impaired autonomic regulation as a mechanistic pathway through which CKD increases cardiovascular risk. Yet, few interventions have been tested to directly improve neural control of circulation in this high-risk population.

Isometric handgrip (IHG) exercise has emerged as a simple, time-efficient, and well-tolerated strategy for BP management (Edwards et al., 2024; Edwards et al., 2023; M et al., 2020; Millar et al., 2013; Millar et al., 2014), as chronic IHG training has been show to lower resting BP more effectively than some conventional exercise modalities (Edwards et al., 2023). Additionally, studies with acute sessions of IHG have shown improvements in autonomic function. For example, Teixeira et al. (Teixeira et al., 2018) demonstrated that one acute session of IHG exercise was capable to enhance cBRS in healthy young adults. Given that patients with CKD present with impaired baroreflex control and heightened BPV, understanding the effects of single session of IHG exercise on these regulatory mechanisms is of particular clinical importance. However, whether these acute autonomic and hemodynamic adaptations to IHG observed in healthy individuals extend to patients with CKD remains unknown.

Therefore, the present proof-of-concept study investigated the acute effects of a single session of IHG exercise on cBRS and beat-to-beat BPV in patients with CKD. We hypothesized that a single session of IHG would acutely enhance cBRS and attenuate BPV, thereby revealing a novel non-pharmacological strategy to improve cardiovascular reflex control in a high-risk population (Go et al., 2004).

## 2. Methods

### 2.1. Ethical approval

All study procedures were approved by the Emory University Institutional Review Board (IRB00009157) in accordance with the Declaration of Helsinki. Written informed consent was obtained for all study participants before participation.

### 2.2. Participants

We enrolled 21 participants with CKD stages III-IV (Table 1), including 9 males and 12 females. The individuals were recruited from Emory University clinics and the Atlanta VA Health Care System for participation in this study. All participants were inactive, defined as engaged in less than 20 min twice per week of self-reported physical activity, and had stable kidney function (no greater than a decline of eGFR of 1 cc/min/1.73 m2 per month over the prior 3 months). Exclusion criteria included uncontrolled hypertension (BP > 160/90 mmHg), vascular disease, use of clonidine, clinical evidence of heart failure or active heart disease determined by history, electrocardiogram (ECG) or echocardiogram, ongoing illicit drug use, alcohol use >2 drinks/day within the past 12 months, diabetic neuropathy, severe anemia (hemoglobin <10 mg/dL), and pregnancy or plans to become pregnant, and engagement in ≥20 min of structured physical activity on two or more days per week, as assessed by the International Physical Activity Questionnaire.

### 2.3. Protocol

**Fig. 1.**
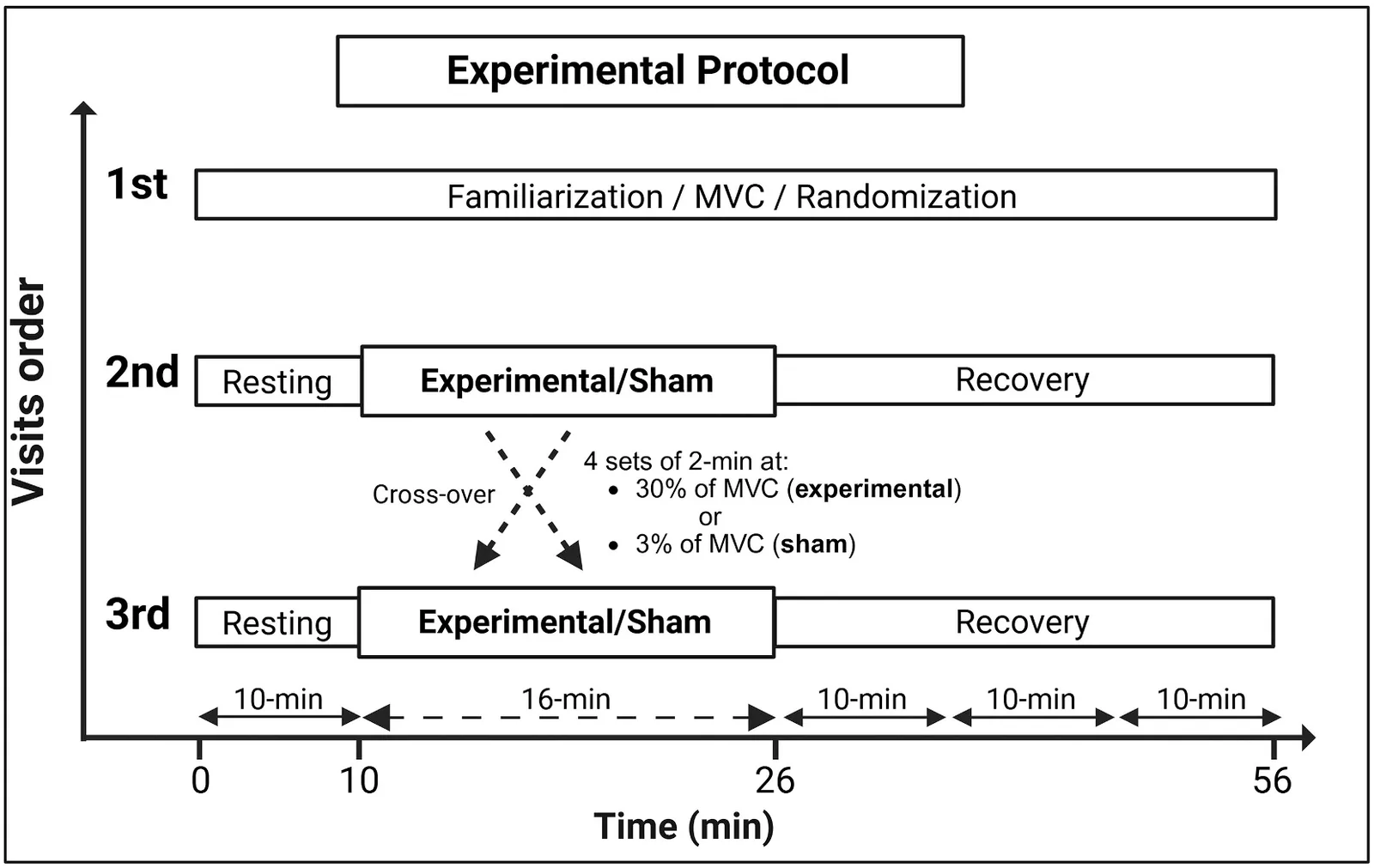
Illustration of the experimental protocol. Each participant completed three visits. The first visit served to familiarize participants with both experimental and sham conditions and for the assessment of maximal voluntary contraction (MVC). The second and third visits were then conducted in a randomized, crossover design. Data analyses were performed using 10-min windows during baseline rest and post-exercise recovery for 30 min. Created in BioRender. https://BioRender.com/d91wznw

The study used a randomized, sham-controlled, crossover, single-blind design, in which each participant completed both the experimental and sham interventions during two study visits separated by 36–72 h in random order (Fig. 1). Randomization and counterbalancing were performed using a computer-generated sequence. A random number list was generated in Excel, which was then used to assign the intervention order. Participants were allocated sequentially according to their enrollment order, following the pre-generated randomization list. The investigator performing data analysis was blinded to the intervention. After obtaining written informed consent, baseline brachial BP and basic metabolic panel were obtained. Participants completed three to five maximal voluntary contractions (MVC), each separated by 1 min of rest, in the dominant hand using a Grip Force Transducer (MLT004/ST; ADInstruments, Bella Vista, NSW, Australia). The highest effort was designated MVC. All participants underwent a familiarization session with the equipment and procedures prior to the experimental protocol to ensure proper execution of the maneuvers. During this session, participants performed at least two sets of 2-min static contractions at 30% (experimental) or 3% (sham) MVC on a separate day. Before each study visit, participants were instructed to abstain from food for at least 4 h and caffeinated beverages for 12 h and exercise and alcohol for a minimum of 24 h. The participants took their regularly scheduled medications similarly before each visit.

After instrumentation, the participants were seated at 90° of hip and knee flexion and rest for 5 min for hemodynamic stabilization, then an additional 10-min period was used to collect baseline measurements. All procedures were performed at an ambient controlled room temperature of ~24 °C. A handgrip dynamometer was interfaced with a computer so that real-time force output feedback could be displayed visually to the participants (Powerlab 16/35; ADInstruments, Bella Vista, NSW, Australia). The protocol consisted of 2 min of IHG exercise in the dominant hand followed by 2 min of recovery (4 min total per set). During the study visit, participants performed 4 sets of isometric contractions at 30% (experimental) or 3% (sham) MVC (16 min total). After completion of exercise, patients were monitored for a 30 min recovery period. Participants returned for a second study visit within 72 h and performed the same protocol with the alternate condition (experiment vs sham). The order of experimental vs sham was randomized and counterbalanced.

### 2.4. Measurements

Continuous heart rate (HR) was measured by a 3-lead ECG (Bioamp System, ADInstruments) and beat-to-beat BP was measured non-invasively by finger photoplethysmography (Nano System, ADInstruments). The finger cuff was kept at heart level during the experiment. Brachial arterial BP was also measured using an automated digital sphygmomanometer (Omron, HEM-907XL, Omron Healthcare, Kyoto, Japan) for absolute measures of BP and to confirm finger measurement accuracy. Respiratory pattern was monitored using a strain-gauge belt placed in a stable position around the abdomen (TN1132/ST; ADInstruments) to ensure that participants kept similar breathing rate before and after exercise and that they did not perform any Valsalva maneuvers during exercise. These signals were recorded at a 1000 Hz sampling rate and stored for offline analysis (PowerLab 16/35, software LabChart 8; ADInstruments). Rating of perceived exertion (RPE) was obtained at the end of exercise using the Borg scale.

### 2.5. Spontaneous cardiac baroreflex sensitivity (cBRS)

The cBRS contributes to the short-term BP control through reflex modulation of autonomic neural activity directed toward the heart (Parati et al., 2001). cBRS was assessed using sequence technique. Briefly, this technique considers progressive increases (up sequences, cBRS_up_) or decreases (down sequences, cBRS_down_) in systolic BP (≥1 mmHg) followed by a progressive lengthening or shortening in RR interval (≥1.0 ms) of three or more heartbeats respectively The slope of the relationship between SBP and RR intervals for all sequences was identified using linear regression analysis, with a minimum *R*^2^ value of 0.85 and 0 beat delay was add to the analysis (CardioSeries v2.4). (Jeong et al., 2024; Sabino-Carvalho et al., 2020; Teixeira et al., 2018). The cBRS for up (cBRS_up_) and down (cBRS_down_) sequences separately, as well as the total (cBRS_all_) sequences detected were analyzed and reported.

### 2.6. Measures of variability

#### 2.6.1. Blood pressure variability (BPV)

Parameters of variability included the standard deviation (SD), the coefficient of variation [(SD/mean) × 100], and the average real variability (ARV) which was calculated using the following formula:

$$ARV=\frac{1}{N\;-\;1}\sum_{k=1}^{N-1}\left|BP_{k+1}-BP_{k}\right|$$

where N denotes the number of BP measurements, k denotes the chronological order of those measurements, as previously reported (Fernandes et al., 2024; Guerrero et al., 2025a; Mena et al., 2005; Sabino-Carvalho et al., 2023b; Teixeira et al., 2022). These parameters were quantified for systolic BP and diastolic BP.

#### 2.6.2. Heart rate variability (HRV)

The ECG data collected was first analyzed using the LabChart HRV module (LabChart Pro v8.1.11 with HRV Module; ADInstruments). The ECG signal was inspected to remove motion artifact-contaminated cardiac cycles from analysis (Gardner et al., 2020). Variables in the time domain were determined, specifically the standard deviation of NN intervals (SDNN) and the root of mean square of successive differences in RR intervals (RMSSD). Fast Fourier transformation was used to acquire power spectral density data, which was analyzed at low frequency (LF) and high frequency (HF) classification values of 0.04–0.15 Hz and 0.15–0.40 Hz, respectively. Spectral components were expressed as normalized units (nu) (Force, 1996).

### 2.7. Statistical analysis

Sample size calculation was completed for the primary variable, cBRS (G*Power 3.1 Dusseldorf University, Dusseldorf, Germany), based on a mean difference of 18% in cBRS after IHG exercise (Teixeira et al., 2018), with a *P*-value set at 0.05 and power set at 0.80. The resultant calculation yielded an estimated sample size of 20 participants. All variables were continuously obtained, and data were averaged and analyzed over a 10-min period for each time point. Normality of each dependent variable was confirmed using the Shapiro–Wilk test and non-parametric test was used when appropriate. Statistical comparisons of physiological variables were made by a General Linear Model for repeated measures with Bonferroni post hoc correction for multiple comparisons in which condition (IHG and Sham) and time (rest, 10-, 20- and 30-min post exercise) were the main factors. The Greenhouse-Geisser’s correction was used to adjust ANOVA results whenever sphericity was violated in the Mauchly’s test. The primary outcome was cBRS, and secondary outcomes were BP, BPV and HRV indexes. Comparisons between conditions were made using paired Students’ *t*-tests. Analyses were conducted using IBM SPSS 26 (SPSS, Chicago, IL). The figures were generated in GraphPad Prism version 8 (Boston, MA, USA). Data are presented as means ± SEM unless otherwise stated and statistical significance was set at *P* ≤ 0.05.

## 3. Results

Baseline characteristics are displayed in Table 1. There were no differences in the number of hours of sleep before the experimental versus sham visits (7 ± 1 vs. 7 ± 1 h; *P* = 0.154). The rate of perceived exertion was higher during the experimental condition (5 ± 2 vs. 2 ± 1 a.u.; *P* = 0.001) compared to sham condition as expected. The desire to void was similar between visits (2 ± 3 vs. 2 ± 2 a.u.; *P* = 0.454).

There were no differences in baseline BP and HR between visits (Table 2). During the recovery period after the experimental (IHG30%) protocol, HR decreased from baseline rest values throughout the 30-min of recovery (10-min: Δ−2 ± 0.5 bpm; 20-min: Δ−2 ± 0.5 bpm; 30-min: Δ−3 ± 0.6 bpm vs. *P* = 0.027 vs. rest) whereas there was no change in HR after the sham (IHG3%) protocol. Systolic BP slightly decreased during the 30-min recovery period after experimental condition (Δ−5 ± 2 mmHg; *P* = 0.047 vs. rest) while systolic BP increased during the recovery period after sham (20-min: Δ5 ± 1 mmHg; *P* = 0.014 vs. rest). No interaction effects were found for diastolic BP (Table 2). Respiratory rate was not different between or within visits (*P* = 0.964).

Both RMSSD and SDNN increased from baseline rest during the recovery period after the experimental (IHG30%) protocol but not after the sham (IHG3%) protocol. The LF/HF ratio was significantly higher 30-min after sham protocol (Table 3). No interaction effects were found for any other measure of HRV (Table 3). No interaction effects were found for any measure of BPV for systolic (all *P* > 0.210) or diastolic BP (all *P* > 0.269; Table 3).

**Fig. 2.**
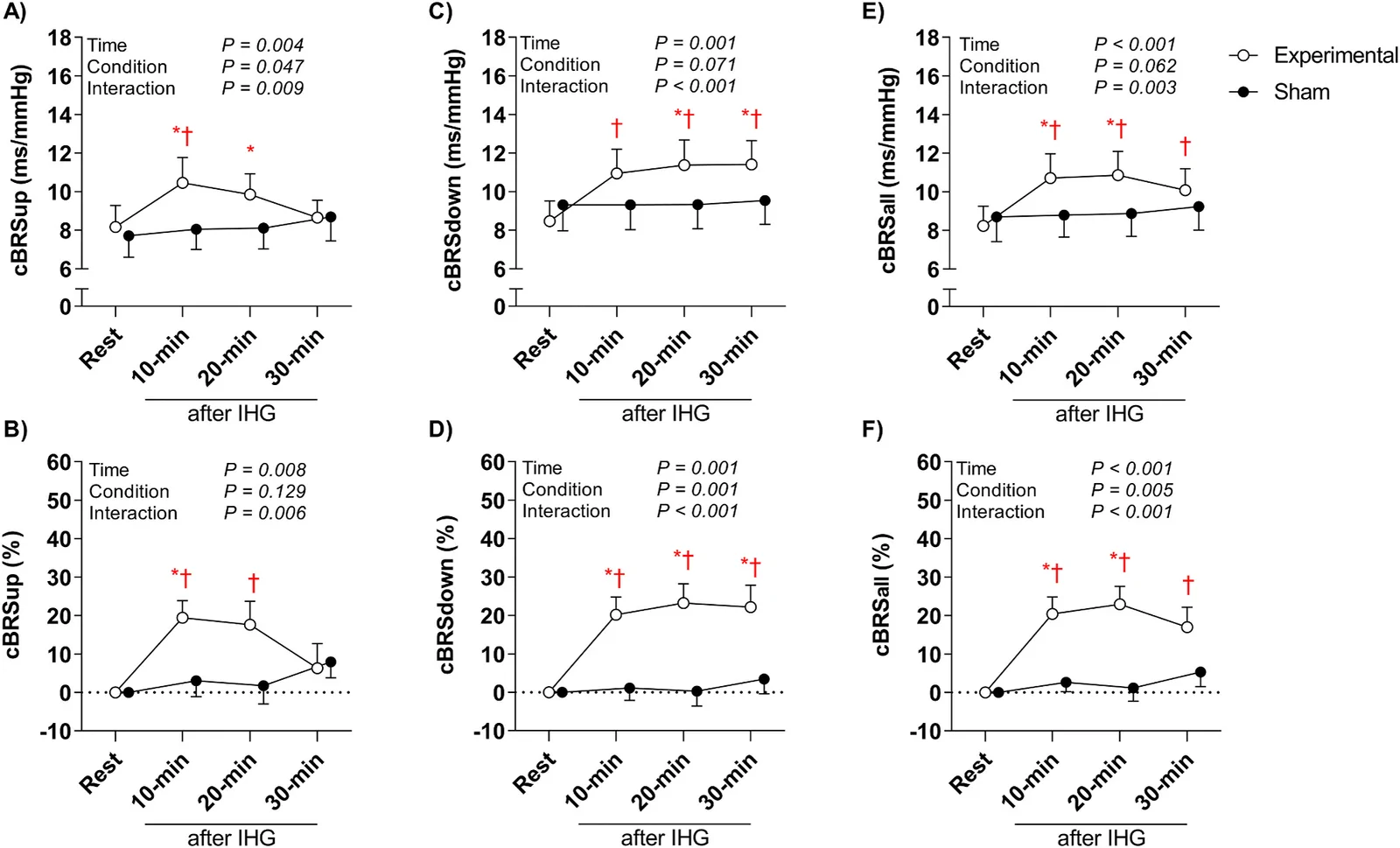
Absolute (top panels) and percent change (bottom panels) in cBRS after a single session of IHG exercise at 30% (experimental) and 3% MVC (sham) in patients with CKD. †*P* < 0.05 vs rest; *P < 0.05 vs sham protocol.

Fig. 2 shows absolute and percent change in cBRS from baseline rest to 10, 20 and 30 min during the recovery period after experimental (IHG30%) versus sham (IHG3%). In the recovery period following the experimental protocol, cBRS_up_ increased at 10- and 20-min of recovery and returned to resting baseline levels by 30-min (Fig. 2A and B). cBRS_down_ remained elevated throughout recovery (Fig. 2C and D). The cBRS_all_ was significantly higher from rest at all recovery time points (Hedges’ g 10-min: 1.44, 95% CI [0.74, 2.13]; 20-min1: 1.51, 95% CI [0.81, 2.21] for percent change from rest), with a sustained but reduced effect at 30 min post-IHG exercise in the experimental condition (Hedges’ g 30-min: 1.02, 95% CI [0.36, 1.68] for percent change from rest). After sham protocol, all variables remained similar to baseline rest levels.

## 4. Discussion

The main novel findings of the present study are that a single session of IHG exercise at 30% of MVC acutely enhances cBRS and increases cardiac parasympathetic modulation as indicated by reductions in HR and increases in RMSSD and SDNN in patients with CKD. These autonomic improvements were accompanied by a modest reduction in systolic BP (5 mmHg) at 30 min post-exercise. In contrast, the sham intervention did not elicit changes in cBRS, HR, or diastolic BP during the recovery period, while systolic BP and LF/HF ratio increased at 30 min post-sham exercise. Importantly, despite improvements in baroreflex function and vagal modulation, no acute changes in beat-to-beat BPV were observed, suggesting that acute improvements in cardiac baroreflex function may not be sufficient to translate into immediate changes in BPV. Collectively, these findings suggest that even a single session of IHG exercise at 30% of MVC can acutely improve cardiac autonomic regulation and transiently reduce systolic BP in CKD without immediately altering BPV.

Our findings extend prior work in healthy populations which demonstrated that acute IHG increases cBRS and vagal modulation (Teixeira et al., 2018). Similar to studies in young healthy adults (Teixeira et al., 2018), the present study confirms that IHG exercise can acutely enhance the sensitivity of arterial baroreflex control of HR (overall improvement of 21 ± 5%) in a chronic disease population. Specifically, we demonstrate for the first time that these acute neural adjustments also occur in patients with CKD, a population characterized by impaired baroreflex function and heightened cardiovascular risk (Matsushita et al., 2022; Sabino-Carvalho et al., 2023b; Sabino-Carvalho et al., 2024). The observed reductions in HR and increases in RMSSD and SDNN indicate that the improvements in cBRS are likely mediated by enhanced cardiac parasympathetic modulation (Edwards et al., 2024). Overall, our findings align with prior evidence suggesting that improvements in baroreflex function after acute isometric exercise are largely vagally mediated (Edwards et al., 2024).

Our group has investigated lifestyle and stress reduction interventions such as exercise that might improve autonomic function in CKD. We recently demonstrated that 12 weeks of aerobic exercise cycling training ameliorated the increase in sympathetic activity over time in older, sedentary patients with CKD (Jeong et al., 2023) but did not improve cardiac parasympathetic modulation (Sabino-Carvalho et al., 2025) or BPV (Sabino-Carvalho et al., 2023a). These findings suggest that aerobic exercise training may modulate sympathetic activity without necessarily altering baroreflex sensitivity although, the direct effects of aerobic exercise on baroreflex function in CKD have not yet been tested. In contrast, our results indicate that isometric exercise may exert greater beneficial effects on baroreflex control in this population. However, whether long-term IHG exercise training can induce sustained improvements in BRS and parasympathetic adaptations remains unexplored in CKD.

Interestingly, although we observed a robust acute improvement in cBRS after IHG exercise, this improvement was not accompanied by reductions in beat-to-beat BPV. This contrasts with prior observations in CKD and other populations, where lower cBRS was associated with higher BPV, suggesting a mechanistic link between impaired baroreflex function and BP fluctuations (Cowley Jr. et al., 1973; Martinka et al., 2005; Miao and Su, 2002; Miao et al., 2006; Sabino-Carvalho et al., 2023b). A recent study also demonstrated that a single IHG session does not acutely alter beat-to-beat BPV in healthy young adults (Guerrero et al., 2025b), even after improvements in cBRS (Teixeira et al., 2018). One possible explanation is that acute improvements in cBRS primarily reflect vagal modulation of HR, which may not be sufficient in the short term to affect the complex interplay of cardiac output, vascular resistance, and central autonomic regulation that determines BPV (Parati et al., 2018). Additionally, BPV may be more influenced by structural vascular factors, sympathetic tone and the sympathetic baroreflex (Young et al., 2020) that require repeated or chronic interventions (Edwards et al., 2024; Edwards et al., 2023). These findings suggest that while acute improvements in cBRS are achievable, longer-term or more intensive interventions may be necessary to modulate BPV in patients with CKD.

The modest 5 mmHg reduction in systolic BP observed 30 min post-IHG is consistent with previous studies demonstrating acute post-exercise hypotension after isometric exercise training (Edwards et al., 2023), even in populations with cardiovascular risk factors. Although small, this reduction is clinically meaningful (Ettehad et al., 2016) given the high cardiovascular risk of patients with CKD and the potential cumulative benefits of repeated sessions. Large-scale meta-analyses have demonstrated that even small decreases in systolic BP translate into substantial reductions in cardiovascular risk (Ettehad et al., 2016). Additionally, the recent hypertension guidelines also highlights that modest BP reductions achieved through lifestyle or behavioral interventions can yield significant population-level benefits (Rao et al., 2025). Therefore, the present acute decrease in systolic BP following IHG exercise, if sustained with repeated sessions, could have meaningful implications for cardiovascular risk and hypertension management in individuals with CKD.

The mechanisms underlying the observed improvements in cBRS and cardiac vagal activity are unclear but may result from enhanced afferent signaling from exercise pressor reflex and central integration within autonomic regulatory circuits (Lehnen et al., 2025a; Lehnen et al., 2025b; Potts et al., 2003). These adaptations may reduce cardiac sympathetic predominance, improve cBRS, and contribute to modest post-exercise reductions in systolic BP (Cho et al., 2025; Edwards et al., 2024; Kirkman and Chavez, 2024; Sprick et al., 2023). While we did not observe acute changes in BPV, it is plausible that repeated IHG sessions could progressively enhance not only the cardiac baroreflex, but also the sympathetic baroreflex that could lead to reductions in BPV as has been previously reported in hypertensive patients after long-term aerobic or resistance exercise training (Caminiti et al., 2021). Therefore, while IHG can rapidly enhance cardiac autonomic control, longer-term or repeated training may be necessary to translate these neural adjustments into measurable reductions in BPV. These findings suggest that isometric handgrip exercise may lead to improvements in baroreflex function that could serve as an important early step toward optimizing cardiovascular stability in CKD population.

### 4.1. Clinical implications

Patients with CKD are at heightened risk of cardiovascular events, partly due to autonomic dysregulation. We demonstrate that a single session of IHG can acutely enhance baroreflex sensitivity and vagal modulation in CKD. Thus, IHG may be a simple, time-efficient, and non-pharmacological strategy to transiently improve cardiovascular autonomic regulation in this population. In addition, isometric handgrip exercise can be performed in a seated manner by individuals that may otherwise have limitations to performing aerobic exercise, thereby increasing access to the benefits of exercise in a larger proportion of patients with chronic disease. Our results offer proof of concept that isometric handgrip training may represent a promising strategy for autonomic control, supporting further investigation in larger clinical studies. Over time, repeated IHG training may contribute to sustained improvements in baroreflex function, reductions in resting BP, and possibly attenuation of cardiovascular risk and should be further studied in longitudinal studies.

### 4.2. Limitations

It is important to consider potential limitations of the present study. First, the sample size was modest, and the cohort included predominantly older adults with CKD stages III–IV which may limit generalizability to earlier CKD stages, end-stage kidney disease or younger populations. Second, BPV was measured acutely after a single session of IHG; the lack of observed changes does not preclude the possibility that chronic IHG training could improve BPV over time. Third, we did not directly measure sympathetic neural activity or vascular function which could provide additional mechanistic insight into the observed hemodynamic responses. Fourth, accounting for the exponential decay relationship between HRV and HR using the correction proposed by Monfredi et al. (Monfredi et al., 2014) did not alter the findings, as the HRV-derived vagal indexes remained statistically significant. Fifth, the study was likely underpowered to detect potential sex differences. Accordingly, we performed exploratory analyses to assess whether responses to the IHG intervention differed between male and female patients. No significant sex-related differences were observed for baseline values or for the responses of cBRS, HRV, BP, or BPV following the intervention (supplementary file). Finally, the study design was limited to 30 min post-exercise. Longer measurements were not performed to avoid signal drift from prolonged single-digit measurements and prevent discomfort. However, longer-term autonomic and hemodynamic recordings may be necessary to fully characterize the duration of these physiologic effects post-acute exercise in CKD.

## 5. Conclusion

In summary, a single session of unilateral IHG exercise at 30% of MVC acutely improves cBRS and enhances cardiac vagal modulation in patients with CKD, with a modest reduction (5 mmHg) in systolic BP. Although beat-to-beat BPV was not altered acutely, this proof-of-concept study suggests that IHG may serve as a practical non-pharmacological intervention to transiently improve cardiovascular autonomic regulation in a high-risk population.

## Supplementary Material

Supplementary Material

## Figures and Tables

**Table 1:** Patient characteristics.

	Patients with CKD (21)

**Descriptive**	
Age, years	61 ± 12
Weight, kg	85 ± 19
BMI, kg/m^2^	29 ± 6
eGFR, mL/min/1.73m^2^	40 ± 9
Right arm dominance, n (%)	19 (90)
MVC, N	302 ± 121
Sex, n of male (%)	9 (43)
Race, n of Black (%)	14 (67)
Hypertension, n (%)	16 (76)
Diabetes, n (%)	4 (19)
**Medication, n (%)**	
ACE	5 (24)
ARB	5 (24)
CCB	8 (38)
Diuretics	9 (43)
Beta blockers	7 (33)
Alpha blockers	1 (5)
Hydralazine	1 (5)
DPP-4 inhibitor	1 (5)
GLP-1 agonist	5 (24)
SGLT-2 inhibitor	7 (33)
Insulin	2 (10)
Sulfonylureas	2 (10)
Statins	11 (52)

Values are mean ± SD. BMI, body mass index; MVC, maximal voluntary contraction; eGFR, estimate glomerular filtration rate. ACEI, angiotensin converting enzyme inhibitor; ARB, angiotensin receptor blocker; CCB, calcium channel blocker; DPP-4, Dipeptidyl Peptidase IV; GLP-1 agonist, glucagon-like peptide-1 receptor agonist; SGLT2, sodium-glucose transport protein 2 inhibitors.

**Table 2:** Hemodynamics variables at rest and after 10, 20, and 30 min of IHG exercise for experimental (30% IHG) and sham (3% IHG) conditions.

	Rest	10-min	20-min	30-min	*P-value*		
					Condition	Time	Interaction

**Hemodynamics**							
**HR, bpm**							
Experimental	67 ± 10	65 ± 10†	65 ± 10†	64 ± 11†			
Sham	67 ± 10	67 ± 11	66 ± 11	66 ± 11	0.313	0.001	0.027
**Systolic BP, mmHg**							
Experimental	117 ± 11	120 ± 16	117 ± 15	113 ± 13†			
Sham	114 ± 13	117 ± 13	119 ± 13†	118 ± 13	0.936	0.007	0.002
**Diastolic BP, mmHg**							
Experimental	69 ± 11	72 ± 16	71 ± 15	69 ± 13			
Sham	68 ± 13	70 ± 13	71 ± 13	72 ± 13	0.912	0.063	0.125
**Respiratory rate, bpm**							
Experimental	15 ± 3	15 ± 3	15 ± 3	15 ± 3			
Sham	15 ± 2	15 ± 3	15 ± 3	15 ± 3	0.519	0.764	0.964

Values are the mean ± SD. IHG, isometric handgrip; HR, heart rate; BP, blood pressure.

† *P* < 0.05 vs rest.

**Table 3:** Blood pressure and heart rate variability at rest and after 10, 20, and 30 min of IHG exercise for experimental (30% IHG) and sham (3% IHG) conditions.

	Rest	10-min	20-min	30-min	*P-value*		
					Condition	Time	Interaction

**Blood pressure variability**							
**Systolic BP**							
ARV, mmHg							
Experimental	2.1 ± 0.7	2.0 ± 0.5	2.0 ± 0.4	2.0 ± 0.5			
Sham	2.2 ± 0.8	2.2 ± 0.6	2.2 ± 0.6	2.4 ± 1.3	0.053	0.037	0.614
SD, mmHg							
Experimental	5.9 ± 1.6	7.9 ± 3.2	7.1 ± 2.2	7.4 ± 3.7			
Sham	7.2 ± 3.6	7.5 ± 4.2	6.6 ± 1.7	7.9 ± 3.4	0.599	0.075	0.210
CV, %							
Experimental	5.1 ± 1.2	6.6 ± 2.7	5.8 ± 1.9	5.9 ± 2.9			
Sham	6.5 ± 3.7	6.7 ± 4.4	5.5 ± 1.5	6.5 ± 2.5	0.455	0.147	0.273
**Diastolic BP**							
ARV, mmHg							
Experimental	1.1 ± 0.7	1.3 ± 1.0	1.4 ± 1.0	1.4 ± 0.8			
Sham	1.0 ± 0.4	1.2 ± 0.4	1.1 ± 0.5	1.2 ± 0.5	0.387	0.001	0.479
SD, mmHg							
Experimental	3.2 ± 1.1	3.8 ± 1.3	3.9 ± 1.4	4.1 ± 1.3			
Sham	3.4 ± 1.4	3.6 ± 1.4	3.4 ± 0.8	4.0 ± 1.2	0.388	0.005	0.355
CV, %							
Experimental	5.0 ± 1.3	6.1 ± 2.1	6.1 ± 2.5	6.1 ± 2.0			
Sham	5.9 ± 2.5	5.9 ± 2.4	5.6 ± 1.2	6.3 ± 1.5	0.862	0.108	0.269
**Heart rate variability**							
**RMSSD, ms**							
Experimental	30 ± 22	39 ± 26†	39 ± 31†	38 ± 30†	-	-	0.038
Sham	31 ± 24	29 ± 20	34 ± 27	32 ± 24	-	-	0.356
**SDNN, ms**							
Experimental	35 ± 12	46 ± 20†	46 ± 19†	47 ± 17†	-	-	0.012
Sham	36 ± 18	39 ± 15	39 ± 20	38 ± 19	-	-	0.196
**LF, nu**							
Experimental	45 ± 25	50 ± 24	39 ± 26	52 ± 24			
Sham	45 ± 25	56 ± 25	48 ± 25	53 ± 25	0.655	0.02	0.537
**HF, nu**							
Experimental	50 ± 21	42 ± 19	45 ± 21	43 ± 21			
Sham	49 ± 21	40 ± 19	46 ± 20	41 ± 18	0.805	0.006	0.960
**LF/HF**							
Experimental	1.6 ± 2.0	1.7 ± 1.7	2.1 ± 2.6	2.0 ± 2.0	-	-	0.121
Sham	1.6 ± 1.9	2.3 ± 2.2†	1.9 ± 2.6	2.5 ± 3.6†	-	-	0.020

Values are the mean ± SD. IHG, isometric handgrip; BP, blood pressure; ARV, average real variability; SD, standard deviation; CV, coefficient of variation; RMSSD, root of mean square of successive differences; SDNN, standard deviation of normal-to-normal intervals; LF, low frequency; HF, high frequency. RMSSD, SDNN, and LF/HF were analyzed using Friedman ANOVA followed by the Wilcoxon test.

† *P* < 0.05 vs rest.

## Appendix A. Supplementary data

Supplementary data to this article can be found online at https://doi.org/10.1016/j.autneu.2026.103446.
