# Supplementary material for: Acute isometric handgrip exercise enhances cardiac baroreflex sensitivity and lowers systolic blood pressure in chronic kidney disease
Source: Auton Neurosci. Author manuscript; Available in PMC 2026 Aug 20. (PMC13491451; doi:10.1016/j.autneu.2026.103446)
Supplement: Supplementary file 1 [file NIHMS2196658-supplement-Supplementary_Material.pdf]

There were no differences in age (58 ± 11 vs. 63 ± 13 years; P = 0.416), weight (89 ± 20 vs. 85 ± 19 kg; P = 0.636), body mass index (29 ± 7 vs. 31 ± 6 kg/m^2 ; P = 0.551) and eGFR (37 ± 7 vs. 41 ± 11 mL/min/1.73m^2; P = 0.351) between males and females, respectively. The maximum voluntary contraction was higher in males versus females (377 ± 117 vs. 234 ± 77 N; P = 0.007). There were no sex differences in blood pressure, heart rate or respiratory during the recovery period after the experimental (IHG30%) protocol (Table 1)

Table 1. Hemodynamics variables at rest and after 10, 20, and 30 min of IHG exercise for experimental (30% IHG) condition between male and female patients.

	Rest	10-min	20-min	30-min	*P-value*		
					**Sex**	**Time**	**Interaction**
**Hemodynamics**							
**HR, bpm**							
Male	66 ± 10	64 ± 9*	64 ± 10	63 ± 10			
Female	68 ± 10	66 ± 11	66 ± 11*	65 ± 12*	0.679	0.001	0.031
**Systolic BP, mmHg**							
Male	120 ± 14	125 ± 14	123 ± 17	117 ± 14			
Female	115 ± 9	116 ± 17	112 ± 13	109 ± 12	0.160	0.004	0.515
**Diastolic BP, mmHg**							
Male	67 ± 12	70 ± 11	69 ± 14	67 ± 13			
Female	71 ± 8	74 ± 15	72 ± 12	71 ± 11	0.457	0.163	0.966
**Respiratory rate, bpm**							
Male	16 ± 3	16 ± 3	16 ± 3	15 ± 3			
Female	15 ± 2	14 ± 3	14 ± 2	15 ± 3	0.896	0.860	0.872

Values are the mean ± SD. IHG, isometric handgrip; HR, heart rate; BP, blood pressure.
† P < 0.05 vs rest.
* P < 0.05 vs sham protocol.

There were no sex differences in blood pressure variability and heart rate variability during the recovery period after the experimental (IHG30%) protocol (Table 2).

Table 2. Blood pressure and heart rate variability at rest and after 10, 20, and 30 min of IHG exercise for experimental (30% IHG) condition between male and female patients.

	Rest	10-min	20-min	30-min	*P-value*		
					Sex	Time	Interaction
**Blood Pressure Variability**							
**Mean BP**							
**ARV, mmHg**							
Male	1.1 ± 0.5	1.1 ± 0.5	1.2 ± 0.5	1.2 ± 0.4			
Female	1.1 ± 0.4	1.3 ± 0.7	1.3 ± 0.6	1.3 ± 0.5	0.687	0.041	0.113
**SD, mmHg**							
Male	3.4 ± 0.9	4.7 ± 1.6	4.6 ± 1.8	4.7 ± 2.1			
Female	4.2 ± 1.2	5.0 ± 2.1	4.8 ± 1.3	5.1 ± 2.1	0.520	0.015	0.860
**CV, %**							
Male	4.2 ± 1.1	5.7 ± 1.9	5.6 ± 2.2	5.7 ± 2.7			
Female	5.1 ± 1.2	6.1 ± 2.5	5.7 ± 1.8	5.8 ± 2.1	0.581	0.061	0.816
**Heart Rate Variability**							
**RMSSD, ms**							
Male	27 ± 15	32 ± 20	31 ± 20	30 ± 21			
Female	32 ± 27	45 ± 30	46 ± 38	44 ± 34	0.320	0.023	0.440
**SDNN, ms**							
Male	34 ± 11	46 ± 24	47 ± 23	46 ± 21			
Female	40 ± 24	45 ± 18	45 ± 17	47 ± 15	0.898	0.016	0.613
**LF, nu**							
Male	55 ± 26	63 ± 20	60 ± 21	60 ± 21			
Female	37 ± 21	39 ± 23	40 ± 27	46 ± 25	0.060	0.267	0.567
**HF, nu**							
Male	41 ± 21	34 ± 19	39 ± 20	38 ± 19			
Female	57 ± 19	48 ± 18	50 ± 21	46 ± 22	0.805	0.006	0.960
**LF/HF**							
Male	2.4 ± 2.7	2.6 ± 2.0	2.8 ± 3.1	2.4 ± 2.1			
Female	0.9 ± 0.9	1.0 ± 0.9	1.4 ± 2.1	1.7 ± 1.9	0.113	0.001	0.085

Values are the mean ± SD. IHG, isometric handgrip; BP, blood pressure; ARV, average real variability; SD, standard deviation; CV, coefficient of variation; RMSSD, root of mean square of successive differences; SDNN, standard deviation of normal-to-normal intervals; LF, low frequency; HF, high frequency.

There were no sex differences in either the absolute or percent change in cBRS from baseline rest to 10, 20 and 30 minutes during the recovery period after experimental (IHG30%; Figure 1).

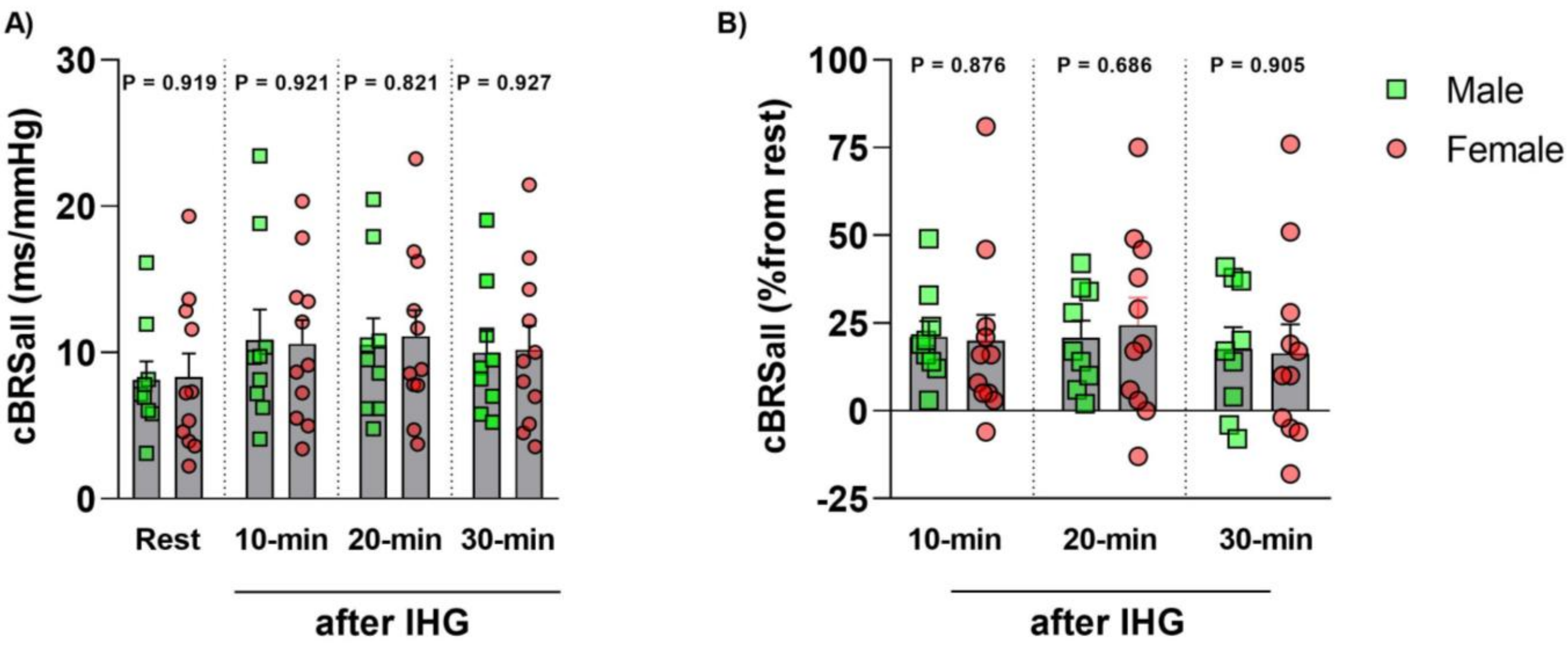

Figure 1. **A.** Absolute and **B.** percent changes in cBRS from rest after a single session of IHG exercise at 30% (experimental) in males (green squares) and females (red circles) patients with CKD.
